# Superhydrophobic, Elastic, and Conducting Polyurethane-Carbon Nanotube–Silane–Aerogel Composite Microfiber

**DOI:** 10.3390/polym12081772

**Published:** 2020-08-07

**Authors:** Taekuk Hong, Sang-Mi Jeong, Yong Kyu Choi, Taekyung Lim, Sanghyun Ju

**Affiliations:** 1 Department of Physics, Kyonggi University, Suwon, Gyeonggi-do 16227, Korea; maegol@naver.com (T.H.); jeongsm@kgu.ac.kr (S.-M.J.); 2 Advanced Institutes of Convergence Technology, Seoul National University, Suwon-si, Gyeonggi-do 16229, Korea; yongkyu2357@gmail.com

**Keywords:** superhydrophobic, elastic, conducting, composite microfiber

## Abstract

Flexible fibers composed of a conductive material mixed with a polymer matrix are useful in wearable electronic devices. However, the presence of the conductive material often reduces the flexibility of the fiber, while the conductivity may be affected by environmental factors such as water and moisture. To address these issues, we developed a new conductive fiber by mixing carbon nanotubes (CNT) with a polyurethane (PU) matrix. A silane ((heptadecafluoro–1,1,2,2–tetra–hydrodecyl)trichlorosilane) was added to improve the strain value of the fiber from 155% to 228%. Moreover, silica aerogel particles were embedded on the fiber surface to increase the water contact angle (WCA) and minimize the effect of water on the conductivity of the fiber. As a result, the fabricated PU-CNT-silane-aerogel composite microfiber maintained a WCA of ~140° even after heating at 250 °C for 30 min. We expect this method of incorporating silane and aerogel to help the development of conductive fibers with high flexibility that are capable of stable operation in wet or humid environments.

## 1. Introduction

High-performance sensors in the form of flexible and stretchable conductive fibers can be woven or stitched into a commercial cloth or applied directly to the human body. The associated textiles (fiber, yarn, or fabrics) also tend to display good resistance to repeated deformation. These properties have been utilized in strain sensors to obtain real-time mechanical feedback in the fields of personal health monitoring, human motion detection, and soft robotics [1,2,3,4]. Such sensors were also applied to the human body to detect ultraviolet (UV) light, chemicals, humidity, and temperature change, either as a portable device or a patch attached to the skin [5,6,7]. Flexible conductive fibers have also attracted interest for use in supercapacitors, interconnects, photovoltaic cells, light-emitting diodes, and artificial skin [8,9,10,11,12]. Despite these potential applications, however, it remains challenging to make flexible conductive fibers with good mechanical properties and durability.

Conductive fibers capable of transmitting electrical signals and electrical energy are often prepared as a composite with polymers to provide mechanical flexibility and strength. Polymer-based nanocomposites have also been considered in order to impart additional desirable properties. Among nanomaterials, carbon nanotubes (CNT) have shown particularly good thermal, mechanical, and electrical properties with a high strength-to-weight ratio. Thus, polymer composites with CNT have been widely applied to not only electronic devices but also in the automobile and aerospace industries [13,14]. When manufacturing the polymer-CNT composites, it is important to form a homogeneous dispersion of CNT within the polymer matrix. Additionally, there should be strong interfacial bonding between these two components to prevent aggregation of CNT in the polymer matrix, which causes a decrease in the mechanical properties reflecting the load transfer efficiency at the interface between CNT and the polymer matrix. For this reason, surface modification of CNT was conducted to facilitate the dispersion of CNT within the polymer matrix [15,16,17]. Despite these efforts, reported polymer-CNT composites tend to show a trade-off relationship between the mechanical properties and the electrical conductivity. Ma et al. fabricated stretchable fibers by mixing multiwall CNT decorated with Ag particles with poly(vinylidene fluoride–co–hexafluoropropylene). The fibers demonstrated a high conductivity of 17,460 S/cm, but the maximum strain at break point was only 60% [18]. Wakuda et al. prepared highly conductive silicone fibers filled with Ag flakes (876 S/cm), which came with the cost of low elasticity (13.8%) [19]. Cheng et al. dip-coated a conductive material on elastic fibers, and found that the coated conductive film was prone to abrasion [20]. Therefore, it is necessary to develop new methods to produce polymer-CNT composites with both superior mechanical properties and high electrical conductivity.

Polyurethane (PU) is widely used in medical supplies as well as in the automotive and aerospace industries due to its excellent mechanical properties. In this study, a conductive PU-CNT (PC) composite microfiber was fabricated through a wet-spinning method. Since a higher CNT content improves the electrical conductivity but degrades the mechanical properties of the microfiber, a silane ((heptadecafluoro–1,1,2,2–tetra–hydrodecyl)trichlorosilane) was added to the PC solution. In the prepared PC-silane (PCS) composite microfiber, the silane formed a strong network that interlinked the polymer chains to increase the mechanical properties. In addition, silica aerogel particles were dispersed in the coagulation bath and embedded on the microfiber surface during fabrication, providing it with hydrophobic properties. The resultant PCS-aerogel (PCSA) composite microfiber displayed robust superhydrophobicity in addition to good elasticity and conductivity.

## 2. Materials and Methods

### 2.1. Fabrication of PC Composite Microfibers

The PC composite microfiber was prepared using a typical wet-spinning process with a coflow wet-spinning machine (Invisible Inc., Gyeonggi-do, South Korea). To prepare the PC solution, 5.7 g of dimethylformamide (DMF, Sigma Aldrich, MO, USA), 3.6 g of tetrahydrofuran (THF, Sigma Aldrich, MO, USA), and 1.3 g of PU powder (Avention) were mixed. Multiwall CNT (diameter ~20 nm; length ~5 μm; purity > 99 wt%, Carbon Nano-material Technology Co.) was added at 0, 0.5, 1.0, 1.5, 2.0, and 2.5 wt% into 10.5 g of the PU solution. (When the CNT concentration was above 2.5 wt%, the mixture became too stiff, and the PU and CNT did not mix.) For smooth mixing, the CNT was first subjected to 60 min of UV–ozone pretreatment (UVO Cleaner, AhTECH Leading Technology Systems Co., Gyeonggi-do, South Korea). After the mixed PC solution was stirred at 300 rpm for 3 h or longer using a magnetic stirrer (MS2026, MTOPS), it was extruded through a single spinneret (18 gauge) made of stainless-steel syringe material, into a coagulation bath filled with deionized (DI) water at an injection speed of 70 µL/min. After the extruded PC microfiber was kept in the bath for 30 min to allow full coagulation, it was taken out and dried in ambient air for 12 h.

### 2.2. Fabrication of PCS Composite Microfibers

A given amount of (heptadecafluoro–1,1,2,2–tetra–hydrodecyl)trichlorosilane (JSI Silicone Co., 2, 4, 6, 8, and 10 µL) was added to 10.5 g of PC solution containing 2.5 wt% CNT. To allow smooth mixing of the solution and facilitate the silane cross-linking reaction, the solution was sonicated for 30 min, followed by stirring at 300 rpm for 15 h. After this, the PCS solution was extruded in the same way as for the PC microfiber.

### 2.3. Fabrication of PCSA Composite Microfibers

The PCSA composite microfiber was prepared using a PCS solution containing 6 µL of silane through the wet-spinning method, but in an aerogel bath instead of DI water bath. The aerogel bath solution was prepared by adding 150 mL of DI water to solutions of 2, 4, 6, 8, and 10 g of aerogel powder (1–20 µm diameter, DAIHAN Scientific Co., Gangwon-do, South Korea) and 50 mL of isopropyl alcohol (IPA, J. T. Baker, Avantor Performance Materials). The PCSA composite microfiber was prepared in the same manner as described above.

### 2.4. Optical, Electrical, Mechanical, and Wetting Properties of Composite Microfibers

The surface morphologies of the composite microfibers were measured using field effect scanning electron microscopy (FESEM, S–4800, Hitachi, Tokyo, Japan ), and chemical mapping was carried out with energy-dispersive X-ray spectroscopy (EDS; 7593–H, Horiba, Kyoto, Japan). Thermogravimetric analysis (TGA; TGA/DSC 1, Mettler Toledo, OH, USA) was used to examine the thermal stability of the microfiber. The mechanical properties were analyzed by a thermal mechanical analyzer (TMA; TMA7000, Hitachi, Tokyo, Japan). The electrical resistance was measured using a digital multimeter (FLUKE–175 EJKCT, Fluke, WA, USA) after forming electrodes using Ag paste at a distance of 10 mm from the composite microfiber. The water contact angle (WCA) of the fabricated composite microfiber was measured using a contact angle analyzer (Phoenix 300, SEO Co., Gyeonggi-do, South Korea).

### 2.5. Water Durability and Thermal Stability of Hydrophobic PCSA Composite Microfibers

The water resistance and thermal stability of the hydrophobic PCSA composite microfibers were examined in water spraying and heating tests, respectively. For the spraying test, a bundle of microfibers with a size of ~25 mm × 30 mm was prepared by uniformly winding the PCSA composite microfibers. Then, water was sprayed onto the whole area at a rate of 600 mL/min through a garden hose nozzle sprayer (Nozzle 5, Takagi, Kokura-Minami-Ku, Japan), from a location 70 mm above the sample. The heating test was carried out by placing the microfiber bundle in a forced convection oven (OF–02GW, JEIO TECH, Daejeon, South Korea) set at 25, 50, 100, 150, 200, and 250 °C for 30 min.

## 3. Results and Discussion

**Figure 1:**
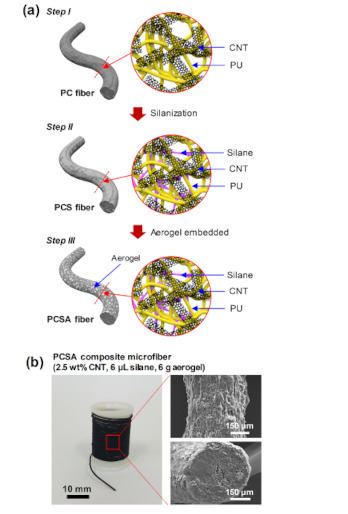
(**a**) Design of the different composite microfibers. (**b**) Left: photograph of the fabricated polyurethane-carbon nanotube-silane-aerogel (PCSA) composite microfiber (carbon nanotubes (CNT) 2.5 wt%, silane 6 μL, aerogel 6 g) wound on a spool. Right: FESEM images of the surface and cross-section of the composite microfiber.

Figure 1a depicts the design of the PCSA composite microfiber with superhydrophobic, elastic, and conductive properties. Three types of composite microfibers were prepared using a similar wet-spinning method. In Step I, conductive and flexible PC composite microfibers were fabricated by combining the flexible PU with highly conductive CNT (top image of Figure 1a). When a large amount of CNT was uniformly distributed throughout the PU matrix, adjacent nanotubes became interconnected to form a conductive CNT network. However, this also reduced the mechanical properties of the fibers. To address this, in Step II a silane was added into the PC solution during fabrication (middle image of Figure 1a). Typically, silane-based coupling agents can create durable bonds between organic and inorganic materials. The selected silane coupling agent, (heptadecafluoro–1,1,2,2–tetra–hydrodecyl)trichlorosilane, has three highly reactive chloride groups that easily form silanols after hydrolysis. These silanol groups can undergo further condensation reactions with other silanol groups to form siloxane linkages, or form hydrogen bonds with other polar groups (–OH, –NH_2_, –SH, –C=O). This means that adding even a small amount of silane to the PC solution led to improved properties via the formation of hydrogen bonds with the urea functional groups of PU to cross-link the polymer chains [21,22,23,24].

For the conductive and flexible composite microfibers used in chemical, UV, and strain sensors, water or moisture in the external environment may cause interference or malfunctions in the sensors [25,26]. To achieve consistent performance, these fibers need to possess better resistance to water and moisture. However, the silane alone did not impart superhydrophobicity to the PCS composite microfiber, since the latter had a low WCA of up to 73.1°. To further improve the resistance to water, in Step III a hydrophobic aerogel was dispersed in water-IPA mixed solution and used as a coagulation bath for the microfiber (bottom image of Figure 1a). During this process, aerogel particles became attached to the surface of the incompletely coagulated PCS composite microfiber. Thanks to this porous and superhydrophobic silica aerogel, the microfiber surface showed a much higher WCA value of ~149.2°. This PCSA composite microfiber (2.5 wt% CNT, 6 μL silane, 6 g aerogel) had a uniform diameter of about 480 ± 20 µm (Figure 1b).

**Figure 2:**
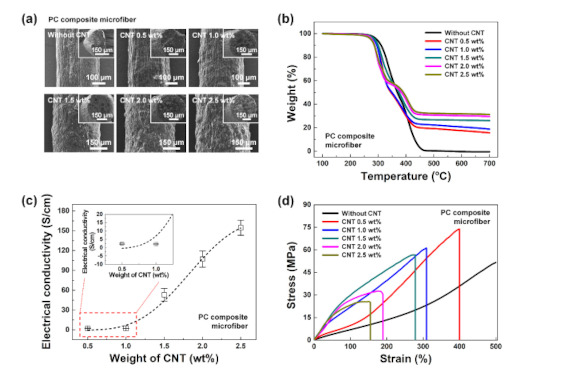
Morphology, thermal, electrical, and mechanical properties of PC composite microfibers with different CNT contents (0, 0.5, 1.0, 1.5, 2.0, and 2.5 wt%): (**a**) FESEM images, (**b**) TGA curves, (**c**) conductivities, and (**d**) stress–strain curves.

In general, in a composite fiber of polymer and CNT, the polymer serves as a matrix with unique properties such as high elasticity, impact resistance, and flexibility [27,28,29]. In this study, PU, which has high elongation and high elasticity, was dissolved at 12 wt% in a mixed solvent of THF and DMF at a 12:7 ratio. Then, highly conductive CNT (0.5–2.5 wt%) was uniformly dispersed in this solution before the PC composite microfibers were produced using a wet-spinning method. It has been reported that during 60 min of UVO treatment, quinine, ester, and hydroxyl functional groups can be formed on the CNT surface without affecting the aspect ratio, making the surface more hydrophilic and facilitating uniform dispersion of CNT in the PU [30]. Figure 2 shows the morphology and thermal, electrical, and mechanical properties of PC composite fibers with different CNT contents. From the FESEM images in Figure 2a, it can be seen that as the CNT content increased, the fiber diameter consistently increased from ~330 (without CNT), ~350 (0.5 wt%), ~380 (1.0 wt%), ~400 (1.5 wt%), ~440 (2.0 wt%), to ~470 μm (2.5 wt%). Furthermore, a higher CNT content means more nanotubes are exposed on the surface, leading to a rougher texture, as shown in the images.

Figure 2b depicts the thermal characteristics of the PC composite microfibers. Without adding CNT, the degradation of PU occurred between ~250–450 °C. Meanwhile, the decomposition of the PC composite fiber was not complete at above 450 °C. It is well known that CNT does not decompose and remains residual at temperatures above 450 °C due to the high thermal stability of CNT. As the CNT content increased, the residual weight of the composite microfibers at 700 °C increased from 0, 15.6, 18.8, 26.0, 29.5, to 31.3% when the CNT content was 0, 0.5, 1.0, 1.5, 2.0, and 2.5 wt%, respectively.

In general, the electrical conductivity of an object is determined by the specific resistance of the conductive materials, the conduction path formed by contact between conductive materials, the junction resistance, and the electron tunneling between noncontact materials. Electron tunneling resistance strongly affects the overall resistance in composites of insulating PU and conducting CNT in particular, because the thin polymer layer between the nanotubes prevents direct contact between them. Thus, an increased CNT content in the composite microfiber leads to a shorter distance between the nanotubes and smoother conduction paths. Additionally, as the minimum distance between a random pair of nanotubes decreases, an electrical-percolation network is formed, increasing the conductivity by decreasing the minimum distance for electron tunneling. Figure 2c shows the electrical conductivity of the PC composite microfibers with different CNT contents. As the CNT content increased, the conductivity gradually increased to 2.4 ± 0.3, 2.2 ± 0.3, 53.1 ± 9.5, 107.1 ± 12.3, and 154.6 ± 11.4 S/cm (0.5, 1.0, 1.5, 2.0, and 2.5 wt%, respectively).

Figure 2d shows the stress–strain curves of the PC composite microfibers. In the pristine PU microfiber without any CNT, a strain of more than 500% was observed, and the microfiber did not break even at the maximum elongation. As the CNT content increased to 0.5, 1.0, 1.5, 2.0, and 2.5 wt%, the stress values decreased to 73.7, 61.1, 56.7, 30.6, and 25.1 MPa, respectively, and the strain values also decreased to 400, 309, 278, 189, and 155%. The addition of powder or particles to a flexible polymer typically leads to decreased flexibility in the microfibers by inhibiting the interaction between polymer chains [31,32]. To ameliorate this problem, it is necessary to add another material to the composite. By using a strong network that interlinked the polymer chains formed by silane, the reduction in mechanical properties due to the aggregation of CNTs while maintaining high conductivity was compensated.

**Figure 3:**
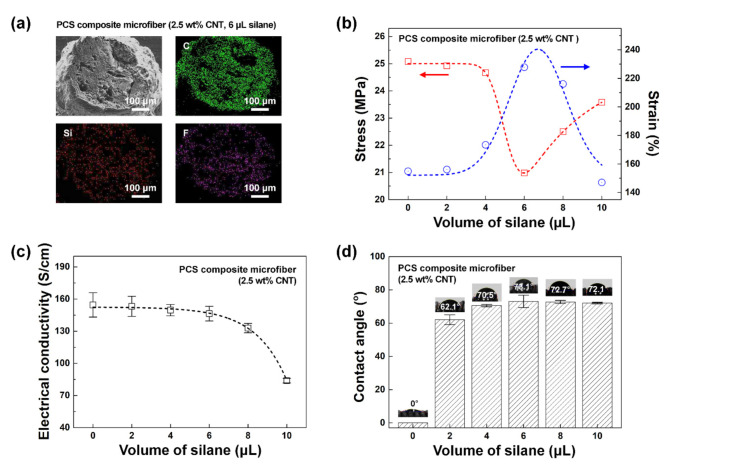
Optical, mechanical, electrical, and wetting properties of PCS composite microfibers. (**a**) FESEM and EDS mapping images (2.5 wt% CNT, 6 µL silane). (**b**) Stress–strain curves, (**c**) electrical conductivities, and (**d**) wettabilities as measured for PCS composite microfibers with different silane contents (0, 2, 4, 6, 8, and 10 µL).

To supplement the mechanical properties of the PC composite microfibers, and the stress–strain value in particular, PCS composite microfibers were prepared by adding different amounts of silane (2, 4, 6, 8, and 10 µL) to the PC solution (2.5 wt% CNT). Figure 3a shows the FESEM and EDS mapping images of a representative PCS composite microfiber (6 µL silane). Si and F from the silane were evenly distributed within the composite microfiber. Meanwhile, the diameter of the composite microfiber changed very little upon addition of silane, at ~480 µm.

Figure 3b displays the mechanical properties of PCS composite microfibers with different silane contents. The strain value gradually increased with increasing silane content. When 6 µL of silane was added, the strain reached a maximum of 228%, a ~1.5-fold increase compared to the value without silane (155%), before gradually decreasing at higher silane contents. This suggests that, up to a certain point, adding more silane results in stronger interactions between silane and PU polymer chains (i.e., hydrogen bonding between OH groups on polysiloxane clusters and the N–H and C=O groups on PU chains) or between silane and CNT (hydrogen bonding between OH groups on polysiloxane and OH groups on UVO-treated CNT). As a result, the strong interactions of silane result in the formation of a strong crosslinked network in the matrix, which improved the flexibility of the microfiber. On the other hand, when too much silane was added (> 6 µL), the siloxane-based clusters produced by the condensation of silane molecules inhibited the interactions described above, resulting in decreased mechanical properties [21,22,23,24]. When 0, 2, 4, 6, 8, and 10 µL of silane was added, the stress value of the PCS composite microfiber was 25.1, 24.9, 24.7, 21.0, 22.5, and 23.6 MPa, respectively. Although the stress value reached a minimum when using 6 µL of silane, the relative decrease was only about 15%. Thus, this change did not greatly offset the remarkable improvement in the strain value.

The effect of silane on the electrical conductivity of the PCS composite microfibers was also investigated (Figure 3c). The conductivity was 154.6, 153.2, 149.7, 146.4, 132.9, and 83.9 S/cm when 0, 2, 4, 6, 8, and 10 µL of silane was added, respectively. Upon addition of up to 6 µL silane, the conductivity decreased by only ~5%. The decrease became more significant when excess silane was used, because the presence of too many siloxane-based clusters interfered with the electrical-percolation network formed by CNT within the composite microfiber.

The silane used in this study had hydrophobic fluorocarbon chains, which were expected to make the PCS composite microfiber hydrophobic. Figure 3d shows the wettability of the PCS composite microfibers as characterized by measured WCA. As the amount of silane increased from 0, 2, 4, 6, 8, to 10 µL, the respective WCA values were 0, 62.1, 70.6, 73.0, 72.7, and 72.1°. Thus, adding silane did increase the WCA compared to the PC composite microfiber, which displayed wetting. Nevertheless, the maximum WCA (73.1°) was relatively low, meaning that the PCS composite microfibers had a low degree of waterproofing. To maintain stable sensing characteristics without sensor malfunction in the presence of external water and moisture, a higher level of hydrophobicity would be necessary.

Generally, a surface is considered hydrophobic when it has a WCA ≥ 90° [33]. Therefore, the PCS composite microfiber with a maximum WCA of 73.1° is not truly hydrophobic. To increase the hydrophobicity, we further embedded silica aerogel particles on the surface of the composite microfiber. Silica aerogels are mesoporous materials characterized by low density, low thermal conductivity, high porosity, and a large surface area [34]. This particular aerogel was expected to improve the hydrophobicity when it was embedded on the microfiber surface due to the hydrophobic property of the C–H bonds [35]. To achieve this, a mixed solution containing IPA, DI water, and the aerogel was used for the coagulation bath during the wet-spinning process, whereas only DI water was employed for the PC and PCS composite microfibers. By using the optimal PCS composite solution (2.5 wt% CNT, 6 μL silane) and adding different amounts of aerogel to the bath (2, 4, 6, 8, and 10 g), PCSA composite microfibers were fabricated. Since the microfibers gradually solidified in the coagulation solution, aerogel particles could be effectively embedded onto the microfiber surface. After drying, hydrophobic composite microfibers with strongly bonded aerogel particles on the surface were obtained.

**Figure 4:**
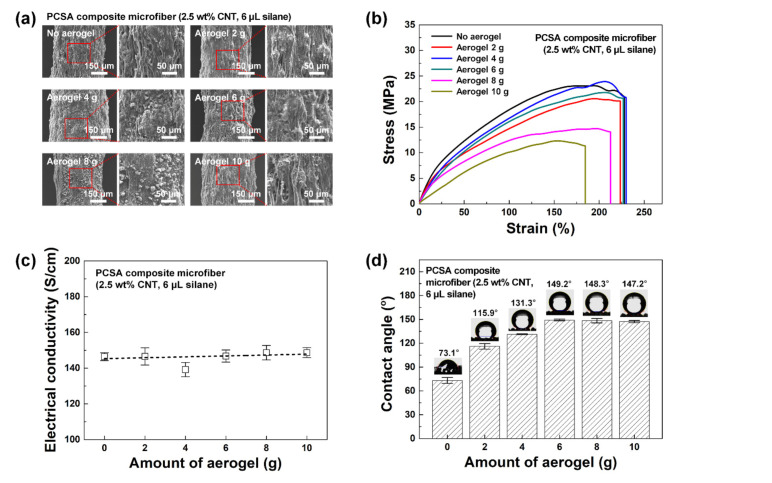
(**a**) FESEM images, (**b**) stress–strain curves, (**c**) electrical conductivities, and (**d**) wettabilities of PCSA composite microfibers (2.5 wt% CNT, 6 μL silane) with different amounts of aerogel (0, 2, 4, 6, 8, and 10 g) added to the coagulation bath during fabrication.

FESEM images of the PSCA composite microfiber surfaces created using different amounts of aerogel (0, 2, 4, 6, 8, and 10 g) are shown in Figure 4a. Regardless of the amount of aerogel in the coagulation solution, the microfibers had a consistent diameter of ~480 μm. When using aerogel in the coagulation bath, aerogel particles were visible on the surface of the fabricated microfibers. The magnified FESEM images (right in Figure 4a) further demonstrate that the amount of embedded aerogel particles was positively correlated with the aerogel content of the bath. The size of the added aerogel particles ranged within 1–20 μm, but the embedded particles had a size of 10 μm or less. As the coagulation solution was not stirred during the wet-spinning process, larger aerogel particles were likely to precipitate rather than remaining dispersed, and thus they were less likely to become embedded. Moreover, even if larger aerogel particles were captured on the surface, they would be more easily removed during the subsequent washing and drying processes due to their weaker bonding with the PU matrix. Therefore, only aerogel particles with a size of 10 μm or less remained embedded on the surface of PCSA composite microfibers.

From the measured stress–strain curves, the PCSA composite microfibers fabricated using less than 6 g aerogel maintained good strain (~227%) and stress (~20.6 MPa) compared to the PCS microfiber (Figure 4b). However, when the amount of aerogel was increased to 8 and 10 g, the strain and stress values decreased to 213% and 14.1 MPa (8 g), and 185% and 11.4 MPa (10 g), respectively. This may be attributed to excess aerogel particles being embedded inside the microfiber in addition to on its surface, which decreased the mechanical properties of the composite microfiber after solidification [36]. In addition, the electrical conductivity data of the PCSA composite microfibers are shown in Figure 4c. Even with the maximum amount of aerogel (10 g), the electrical conductivity was maintained at a high value of ~146.1 S/cm.

Finally, Figure 4d shows the wettability of the different PCSA composite microfibers. The WCA values were 73.1°, 115.9°, 131.3°, 149.2°, 148.3°, and 147.2° when 0, 2, 4, 6, 8, and 10 g of aerogel was used in the coagulation solution, respectively. A saturated WCA value was achieved when 6 g or more aerogel was used. When the WCA was close to 150°, the surface of the PCSA composite microfiber could be termed superhydrophobic. The above results confirmed that the hydrophobicity of the composite microfiber could be effectively improved by incorporating a hydrophobic aerogel in the coagulation bath without impacting the electrical and mechanical properties, provided that the amount of aerogel added did not exceed 6 g.

**Figure 5:**
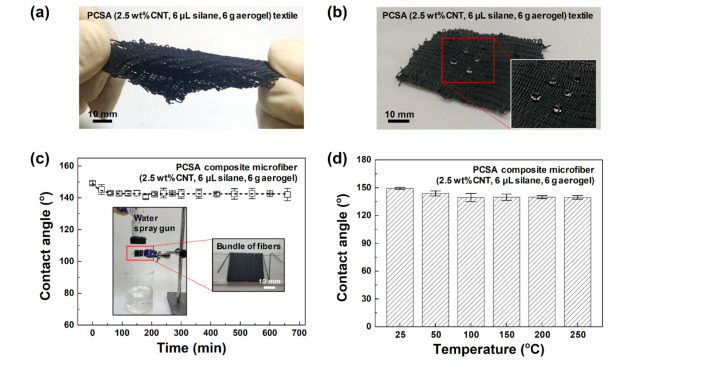
(**a**) Demonstration of elasticity when stress is applied to textile woven from the PCSA composite microfiber (2.5 wt% CNT, 6 μL silane, 6 g aerogel). (**b**) Photograph of the textile when exposed to drops of water. WCA of PCSA composite microfibers after (**c**) water spray and (**d**) heating tests.

The optimal PCSA composite microfiber (2.5 wt% CNT, 6 μL silane, 6 g aerogel) was woven into a textile with a size of 55 mm × 50 mm. Figure 5a demonstrates the high elasticity of the textile. In Figure 5b, water drops maintained their shape without penetrating the textile, since the surface was hydrophobic thanks to the surface-embedded silica aerogel and silane within the microfiber. In Figure 5c, a bundle of the PCSA composite microfibers was subjected to a continuous water spray at 600 mL/min. The original WCA value (149.2°) decreased only slightly, to 145.3° after 30 min and 143.0° after 60 min. Even after 660 min of spraying, the average WCA value remained constant at 142.5°. The initial decrease of WCA from 0–60 min may be explained by the water washing away the loosely bound aerogel particles on the surface. However, this decrease of ~7° amounts to a relative change of merely 4%, and the final WCA maintained was above 140°. Additionally, we found that the hydrophobicity was hardly affected by heat treatment. In Figure 5d, a bundle of the same PCSA composite microfibers was heated to 50–250 °C (50 °C temperature steps) for 30 min. After the thermal treatment, the WCA only slightly decreased, from the initial 149.2° to 143.8° and 139.3° at 50 and 100 °C, respectively, and stayed at about 140° even after heating to 250 °C. Based on these results, we conclude that the hydrophobic property of the fabricated PCSA composite microfiber was maintained with continuous exposure to water or a high temperature of 250 °C.

## 4. Conclusions

In summary, CNT, silane, and silica aerogel were added to a PU matrix to impart high conductivity, good elasticity, and hydrophobicity, respectively, to the fabricated composite microfibers. After adding 2.5 wt% CNT to the PU polymer solution, the PC composite microfiber showed an increased electrical conductivity of 154.6 S/cm, albeit with decreased mechanical properties (stress: 25.1 MPa, strain: 155%). In order to restore the mechanical properties, silane was added to the PC composite solution, and the resultant PCS composite microfiber (2.5 wt% CNT, 6 μL silane) showed a strain value 47% higher than that without silane and improved stretchability. In addition, silica aerogel particles were added to the coagulation bath and were embedded on the microfiber surface after the wet-spinning process, improving the surface hydrophobicity. The optimal PCSA composite microfiber (2.5 wt% CNT, 6 μL silane, 6 g aerogel) displayed a superhydrophobic surface with a maximum WCA of 149.2°, good elasticity with a strain of about 227%, and a high conductivity of 146.8 S/cm. Even after continuous water spraying (600 mL/min, up to 660 min) or heating (50–250 °C for 30 min), the WCA was maintained at about 140°. This conductive composite microfiber may be applied in flexible and wearable fiber-based electronic devices for stable operation in the presence of water or moisture.
